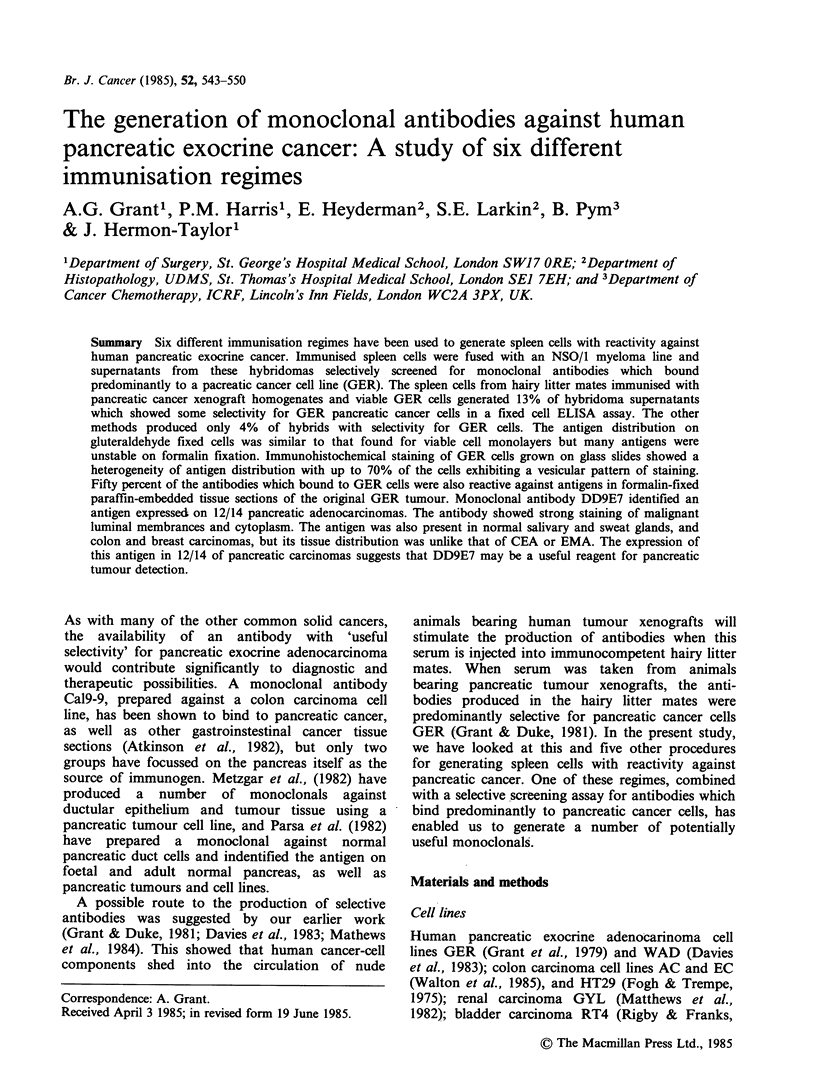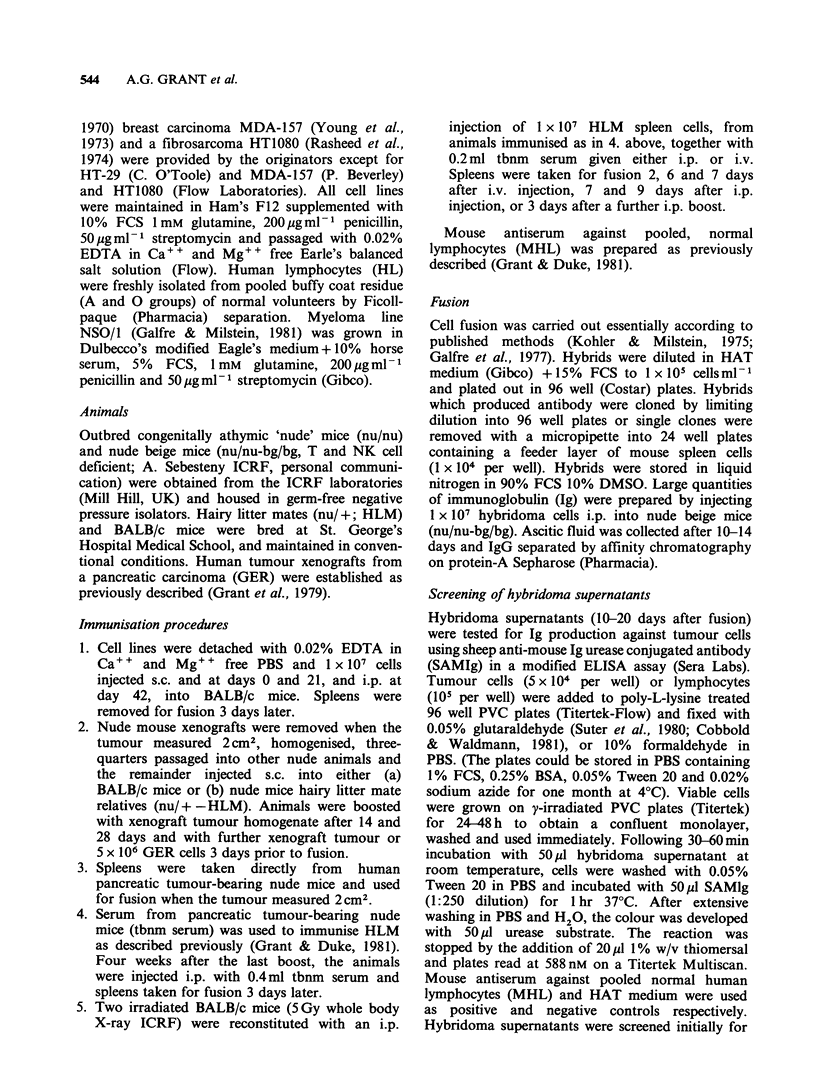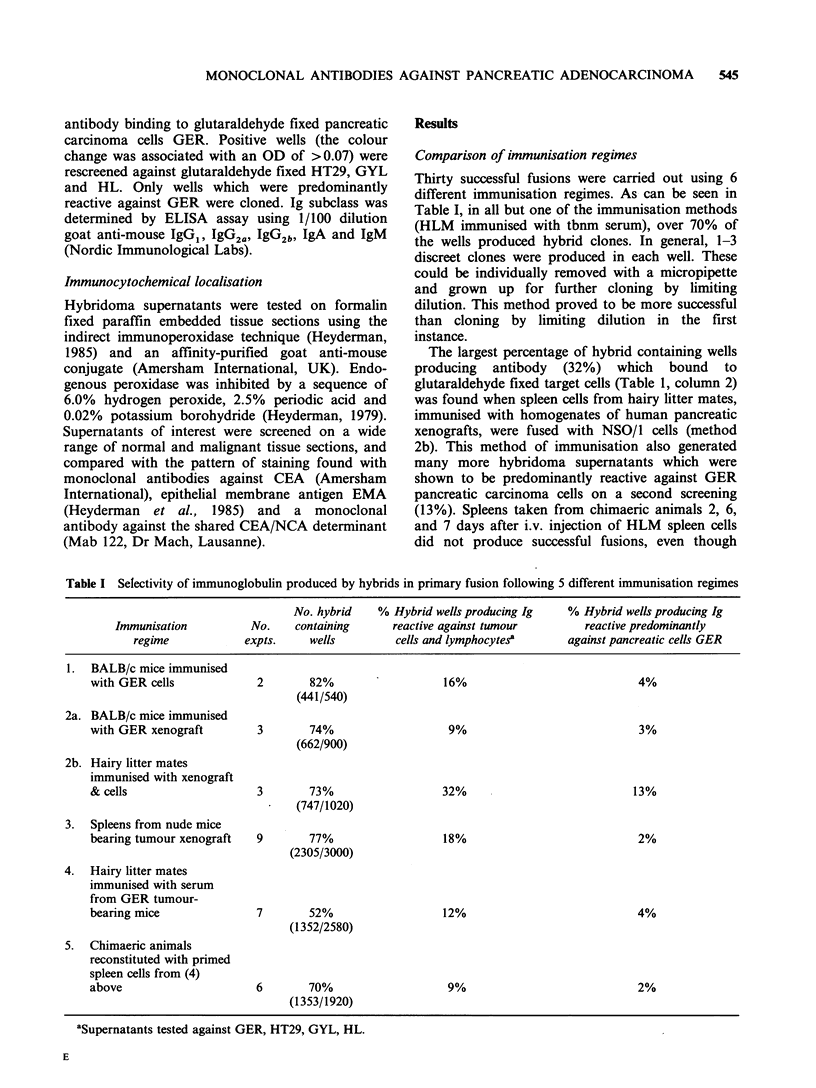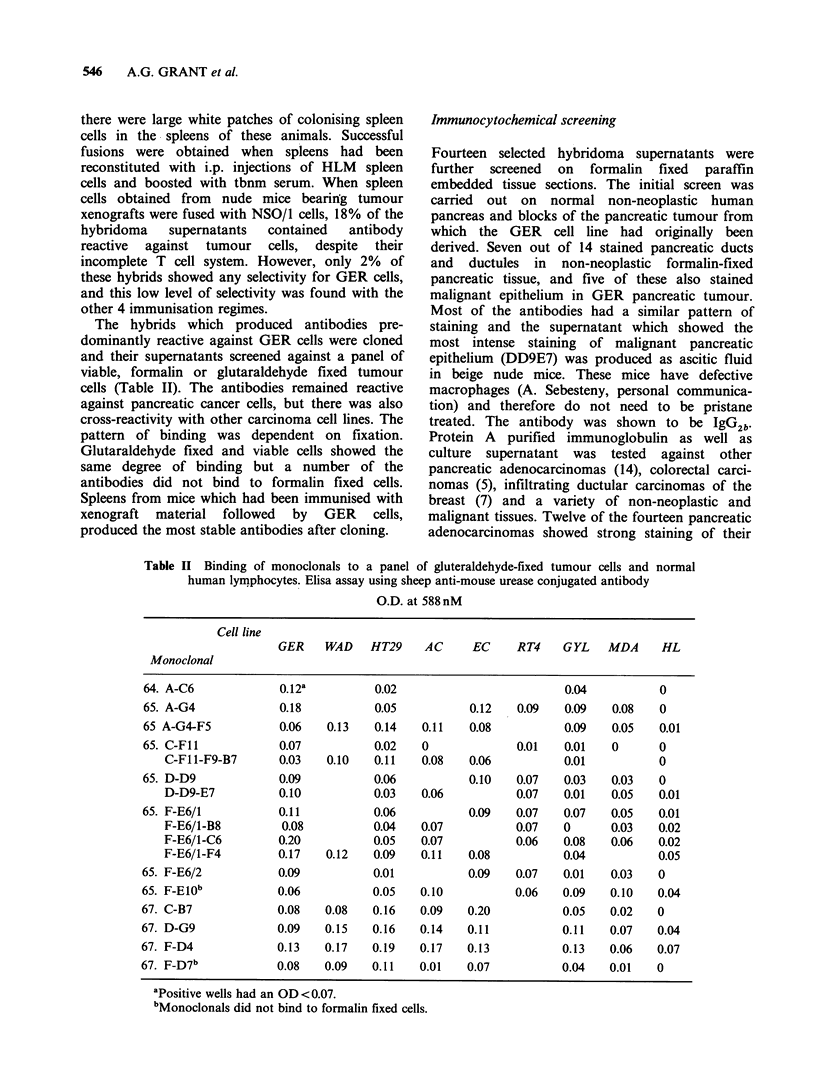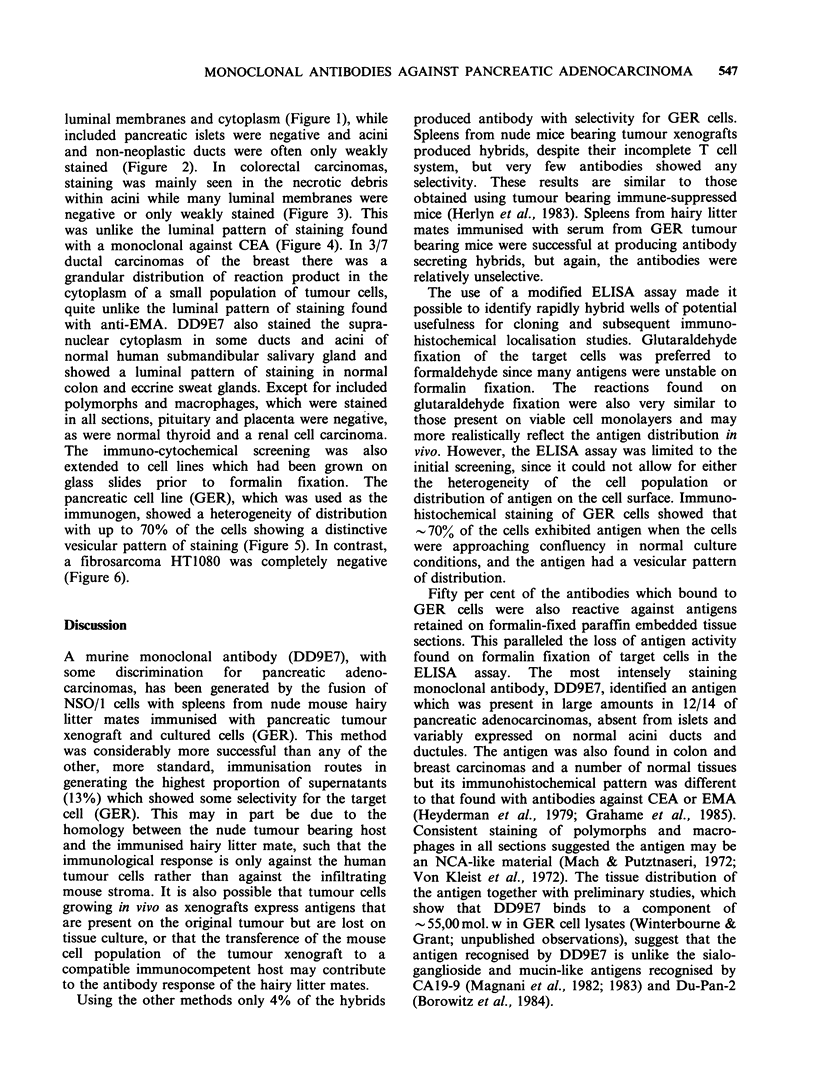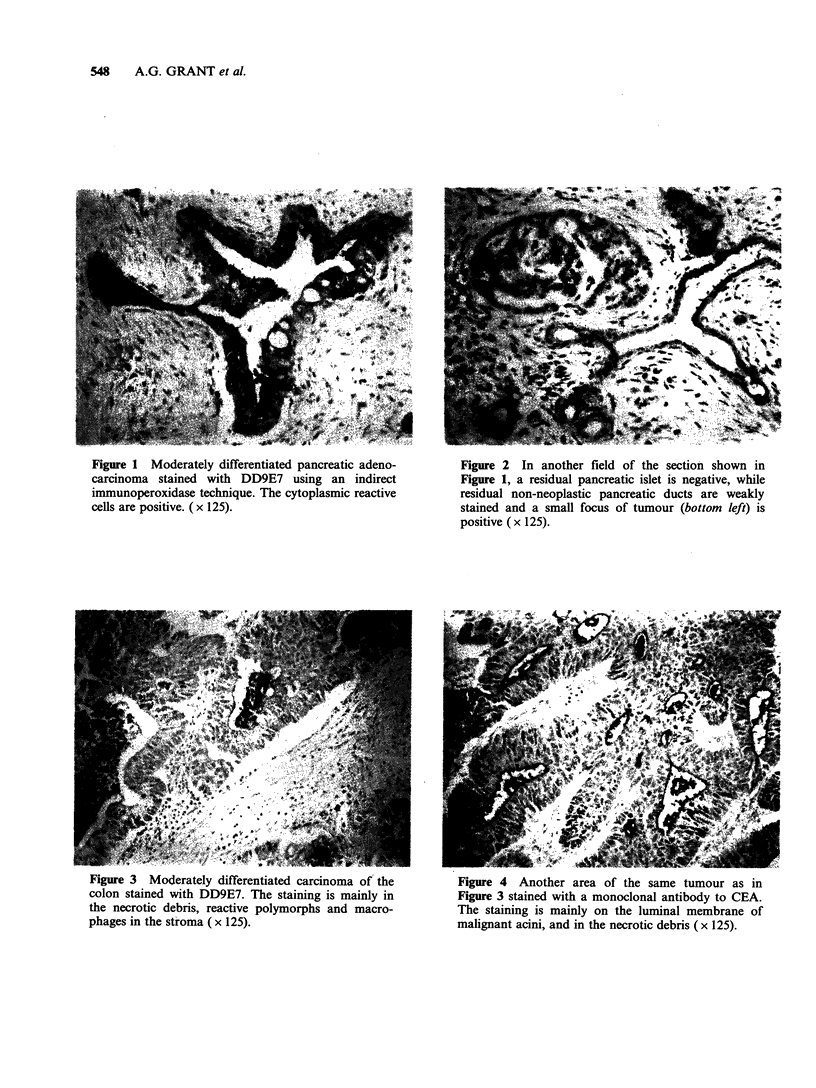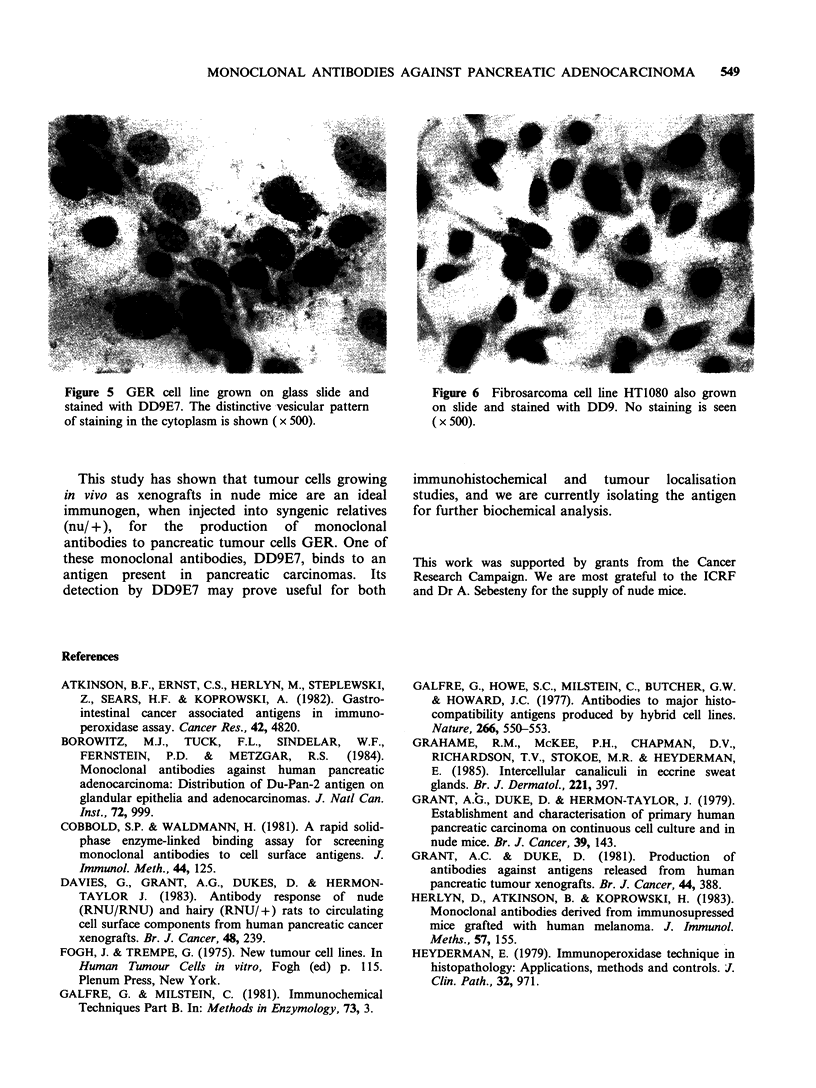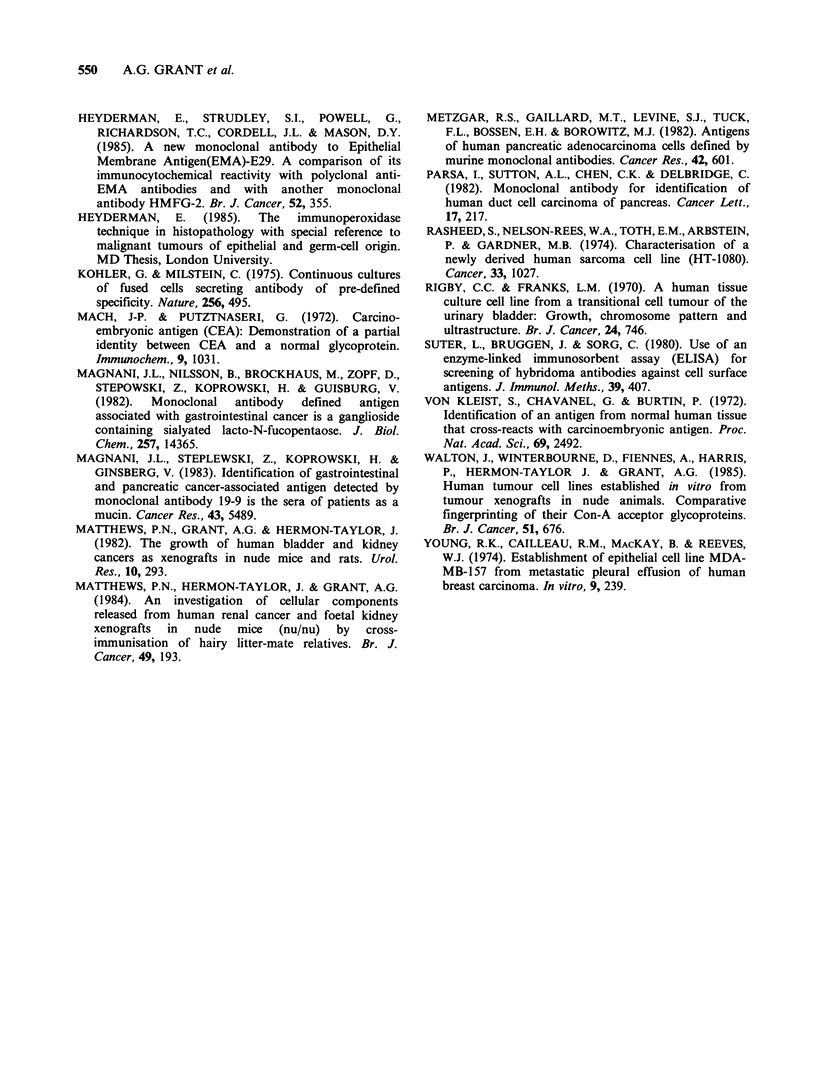# The generation of monoclonal antibodies against human pancreatic exocrine cancer: a study of six different immunisation regimes.

**DOI:** 10.1038/bjc.1985.226

**Published:** 1985-10

**Authors:** A. G. Grant, P. M. Harris, E. Heyderman, S. E. Larkin, B. Pym, J. Hermon-Taylor

## Abstract

**Images:**


*Br. J. Cancer* (1985), **52**, 543–550

### The generation of monoclonal antibodies against human pancreatic exocrine cancer: A study of six different immunisation regimes

A.G. Grant[1], P.M. Harris[1], E. Heyderman[2], S.E. Larkin[2], B. Pym[3] & J. Hermon-Taylor[1]

[1]*Department of Surgery, St. George's Hospital Medical School, London SW17 0RE;* [2]*Department of Histopathology, UDMS, St. Thomas's Hospital Medical School, London SE1 7EH; and* [3]*Department of Cancer Chemotherapy, ICRF, Lincoln's Inn Fields, London WC2A 3PX, UK.*

**Summary** Six different immunisation regimes have been used to generate spleen cells with reactivity against human pancreatic exocrine cancer. Immunised spleen cells were fused with an NSO/1 myeloma line and supernatants from these hybridomas selectively screened for monoclonal antibodies which bound predominantly to a pacreatic cancer cell line (GER). The spleen cells from hairy litter mates immunised with pancreatic cancer xenograft homogenates and viable GER cells generated 13% of hybridoma supernatants which showed some selectivity for GER pancreatic cancer cells in a fixed cell ELISA assay. The other methods produced only 4% of hybrids with selectivity for GER cells. The antigen distribution on gluteraldehyde fixed cells was similar to that found for viable cell monolayers but many antigens were unstable on formalin fixation. Immunohistochemical staining of GER cells grown on glass slides showed a heterogeneity of antigen distribution with up to 70% of the cells exhibiting a vesicular pattern of staining. Fifty percent of the antibodies which bound to GER cells were also reactive against antigens in formalin-fixed paraffin-embedded tissue sections of the original GER tumour. Monoclonal antibody DD9E7 identified an antigen expressed on 12/14 pancreatic adenocarcinomas. The antibody showed strong staining of malignant luminal membrances and cytoplasm. The antigen was also present in normal salivary and sweat glands, and colon and breast carcinomas, but its tissue distribution was unlike that of CEA or EMA. The expression of this antigen in 12/14 of pancreatic carcinomas suggests that DD9E7 may be a useful reagent for pancreatic tumour detection.

As with many of the other common solid cancers, the availability of an antibody with 'useful selectivity' for pancreatic exocrine adenocarcinoma would contribute significantly to diagnostic and therapeutic possibilities. A monoclonal antibody Cal9-9, prepared against a colon carcinoma cell line, has been shown to bind to pancreatic cancer, as well as other gastroinstestinal cancer tissue sections (Atkinson *et al.*, 1982), but only two groups have focussed on the pancreas itself as the source of immunogen. Metzgar *et al.*, (1982) have produced a number of monoclonals against ductular epithelium and tumour tissue using a pancreatic tumour cell line, and Parsa *et al.* (1982) have prepared a monoclonal against normal pancreatic duct cells and indentified the antigen on foetal and adult normal pancreas, as well as pancreatic tumours and cell lines.

A possible route to the production of selective antibodies was suggested by our earlier work (Grant & Duke, 1981; Davies *et al.*, 1983; Mathews *et al.*, 1984). This showed that human cancer-cell components shed into the circulation of nude animals bearing human tumour xenografts will stimulate the production of antibodies when this serum is injected into immunocompetent hairy litter mates. When serum was taken from animals bearing pancreatic tumour xenografts, the antibodies produced in the hairy litter mates were predominantly selective for pancreatic cancer cells GER (Grant & Duke, 1981). In the present study, we have looked at this and five other procedures for generating spleen cells with reactivity against pancreatic cancer. One of these regimes, combined with a selective screening assay for antibodies which bind predominantly to pancreatic cancer cells, has enabled us to generate a number of potentially useful monoclonals.

Correspondence: A. Grant.
Received April 3 1985; in revised form 19 June 1985.

### Materials and methods

### *Cell lines*

Human pancreatic exocrine adenocarinoma cell lines GER (Grant *et al.*, 1979) and WAD (Davies *et al.*, 1983); colon carcinoma cell lines AC and EC (Walton *et al.*, 1985), and HT29 (Fogh & Trempe, 1975); renal carcinoma GYL (Matthews *et al.*, 1982); bladder carcinoma RT4 (Rigby & Franks,

© The Macmillan Press Ltd., 1985

1970) breast carcinoma MDA-157 (Young et al., 1973) and a fibrosarcoma HT1080 (Rasheed et al., 1974) were provided by the originators except for HT-29 (C. O'Toole) and MDA-157 (P. Beverley) and HT1080 (Flow Laboratories). All cell lines were maintained in Ham's F12 supplemented with 10% FCS 1 mM glutamine, 200 $\mu g\,ml^{-1}$ penicillin, 50 $\mu g\,ml^{-1}$ streptomycin and passaged with 0.02% EDTA in $Ca^{++}$ and $Mg^{++}$ free Earle's balanced salt solution (Flow). Human lymphocytes (HL) were freshly isolated from pooled buffy coat residue (A and O groups) of normal volunteers by Ficoll-paque (Pharmacia) separation. Myeloma line NSO/1 (Galfre & Milstein, 1981) was grown in Dulbecco's modified Eagle's medium + 10% horse serum, 5% FCS, 1 mM glutamine, 200 $\mu g\,ml^{-1}$ penicillin and 50 $\mu g\,ml^{-1}$ streptomycin (Gibco).

*Animals*

Outbred congenitally athymic 'nude' mice (nu/nu) and nude beige mice (nu/nu-bg/bg, T and NK cell deficient; A. Sebesteny ICRF, personal communication) were obtained from the ICRF laboratories (Mill Hill, UK) and housed in germ-free negative pressure isolators. Hairy litter mates (nu/+; HLM) and BALB/c mice were bred at St. George's Hospital Medical School, and maintained in conventional conditions. Human tumour xenografts from a pancreatic carcinoma (GER) were established as previously described (Grant et al., 1979).

*Immunisation procedures*

1. Cell lines were detached with 0.02% EDTA in $Ca^{++}$ and $Mg^{++}$ free PBS and $1 \times 10^7$ cells injected s.c. and at days 0 and 21, and i.p. at day 42, into BALB/c mice. Spleens were removed for fusion 3 days later.
2. Nude mouse xenografts were removed when the tumour measured 2 $cm^2$, homogenised, three-quarters passaged into other nude animals and the remainder injected s.c. into either (a) BALB/c mice or (b) nude mice hairy litter mate relatives (nu/+ − HLM). Animals were boosted with xenograft tumour homogenate after 14 and 28 days and with further xenograft tumour or $5 \times 10^6$ GER cells 3 days prior to fusion.
3. Spleens were taken directly from human pancreatic tumour-bearing nude mice and used for fusion when the tumour measured 2 $cm^2$.
4. Serum from pancreatic tumour-bearing nude mice (tbnm serum) was used to immunise HLM as described previously (Grant & Duke, 1981). Four weeks after the last boost, the animals were injected i.p. with 0.4 ml tbnm serum and spleens taken for fusion 3 days later.
5. Two irradiated BALB/c mice (5 Gy whole body X-ray ICRF) were reconstituted with an i.p. injection of $1 \times 10^7$ HLM spleen cells, from animals immunised as in 4. above, together with 0.2 ml tbnm serum given either i.p. or i.v. Spleens were taken for fusion 2, 6 and 7 days after i.v. injection, 7 and 9 days after i.p. injection, or 3 days after a further i.p. boost.

Mouse antiserum against pooled, normal lymphocytes (MHL) was prepared as previously described (Grant & Duke, 1981).

*Fusion*

Cell fusion was carried out essentially according to published methods (Kohler & Milstein, 1975; Galfre et al., 1977). Hybrids were diluted in HAT medium (Gibco) + 15% FCS to $1 \times 10^5$ cells $ml^{-1}$ and plated out in 96 well (Costar) plates. Hybrids which produced antibody were cloned by limiting dilution into 96 well plates or single clones were removed with a micropipette into 24 well plates containing a feeder layer of mouse spleen cells ($1 \times 10^4$ per well). Hybrids were stored in liquid nitrogen in 90% FCS 10% DMSO. Large quantities of immunoglobulin (Ig) were prepared by injecting $1 \times 10^7$ hybridoma cells i.p. into nude beige mice (nu/nu-bg/bg). Ascitic fluid was collected after 10–14 days and IgG separated by affinity chromatography on protein-A Sepharose (Pharmacia).

*Screening of hybridoma supernatants*

Hybridoma supernatants (10–20 days after fusion) were tested for Ig production against tumour cells using sheep anti-mouse Ig urease conjugated antibody (SAMIg) in a modified ELISA assay (Sera Labs). Tumour cells ($5 \times 10^4$ per well) or lymphocytes ($10^5$ per well) were added to poly-L-lysine treated 96 well PVC plates (Titertek-Flow) and fixed with 0.05% glutaraldehyde (Suter et al., 1980; Cobbold & Waldmann, 1981), or 10% formaldehyde in PBS. (The plates could be stored in PBS containing 1% FCS, 0.25% BSA, 0.05% Tween 20 and 0.02% sodium azide for one month at 4°C). Viable cells were grown on $\gamma$-irradiated PVC plates (Titertek) for 24–48 h to obtain a confluent monolayer, washed and used immediately. Following 30–60 min incubation with 50 $\mu l$ hybridoma supernatant at room temperature, cells were washed with 0.05% Tween 20 in PBS and incubated with 50 $\mu l$ SAMIg (1:250 dilution) for 1 hr 37°C. After extensive washing in PBS and $H_2O$, the colour was developed with 50 $\mu l$ urease substrate. The reaction was stopped by the addition of 20 $\mu l$ 1% w/v thiomersal and plates read at 588 nM on a Titertek Multiscan. Mouse antiserum against pooled normal human lymphocytes (MHL) and HAT medium were used as positive and negative controls respectively. Hybridoma supernatants were screened initially for antibody binding to glutaraldehyde fixed pancreatic carcinoma cells GER. Positive wells (the colour change was associated with an OD of >0.07) were rescreened against glutaraldehyde fixed HT29, GYL and HL. Only wells which were predominantly reactive against GER were cloned. Ig subclass was determined by ELISA assay using 1/100 dilution goat anti-mouse $IgG_1$, $IgG_{2a}$, $IgG_{2b}$, IgA and IgM (Nordic Immunological Labs).

*Immunocytochemical localisation*

Hybridoma supernatants were tested on formalin fixed paraffin embedded tissue sections using the indirect immunoperoxidase technique (Heyderman, 1985) and an affinity-purified goat anti-mouse conjugate (Amersham International, UK). Endogenous peroxidase was inhibited by a sequence of 6.0% hydrogen peroxide, 2.5% periodic acid and 0.02% potassium borohydride (Heyderman, 1979). Supernatants of interest were screened on a wide range of normal and malignant tissue sections, and compared with the pattern of staining found with monoclonal antibodies against CEA (Amersham International), epithelial membrane antigen EMA (Heyderman *et al.,* 1985) and a monoclonal antibody against the shared CEA/NCA determinant (Mab 122, Dr Mach, Lausanne).

### Results

*Comparison of immunisation regimes*

Thirty successful fusions were carried out using 6 different immunisation regimes. As can be seen in Table I, in all but one of the immunisation methods (HLM immunised with tbnm serum), over 70% of the wells produced hybrid clones. In general, 1–3 discreet clones were produced in each well. These could be individually removed with a micropipette and grown up for further cloning by limiting dilution. This method proved to be more successful than cloning by limiting dilution in the first instance.

The largest percentage of hybrid containing wells producing antibody (32%) which bound to glutaraldehyde fixed target cells (Table 1, column 2) was found when spleen cells from hairy litter mates, immunised with homogenates of human pancreatic xenografts, were fused with NSO/1 cells (method 2b). This method of immunisation also generated many more hybridoma supernatants which were shown to be predominantly reactive against GER pancreatic carcinoma cells on a second screening (13%). Spleens taken from chimaeric animals 2, 6, and 7 days after i.v. injection of HLM spleen cells did not produce successful fusions, even though

**Table I** Selectivity of immunoglobulin produced by hybrids in primary fusion following 5 different immunisation regimes

| *Immunisation regime* | *No. expts.* | *No. hybrid containing wells* | *% Hybrid wells producing Ig reactive against tumour cells and lymphocytes*[a] | *% Hybrid wells producing Ig reactive predominantly against pancreatic cells GER* |
|---|---|---|---|---|
| 1. BALB/c mice immunised with GER cells | 2 | 82% (441/540) | 16% | 4% |
| 2a. BALB/c mice immunised with GER xenograft | 3 | 74% (662/900) | 9% | 3% |
| 2b. Hairy litter mates immunised with xenograft & cells | 3 | 73% (747/1020) | 32% | 13% |
| 3. Spleens from nude mice bearing tumour xenograft | 9 | 77% (2305/3000) | 18% | 2% |
| 4. Hairy litter mates immunised with serum from GER tumour-bearing mice | 7 | 52% (1352/2580) | 12% | 4% |
| 5. Chimaeric animals reconstituted with primed spleen cells from (4) above | 6 | 70% (1353/1920) | 9% | 2% |

[a]Supernatants tested against GER, HT29, GYL, HL.

there were large white patches of colonising spleen cells in the spleens of these animals. Successful fusions were obtained when spleens had been reconstituted with i.p. injections of HLM spleen cells and boosted with tbnm serum. When spleen cells obtained from nude mice bearing tumour xenografts were fused with NSO/1 cells, 18% of the hybridoma supernatants contained antibody reactive against tumour cells, despite their incomplete T cell system. However, only 2% of these hybrids showed any selectivity for GER cells, and this low level of selectivity was found with the other 4 immunisation regimes.

The hybrids which produced antibodies predominantly reactive against GER cells were cloned and their supernatants screened against a panel of viable, formalin or glutaraldehyde fixed tumour cells (Table II). The antibodies remained reactive against pancreatic cancer cells, but there was also cross-reactivity with other carcinoma cell lines. The pattern of binding was dependent on fixation. Glutaraldehyde fixed and viable cells showed the same degree of binding but a number of the antibodies did not bind to formalin fixed cells. Spleens from mice which had been immunised with xenograft material followed by GER cells, produced the most stable antibodies after cloning.

*Immunocytochemical screening*

Fourteen selected hybridoma supernatants were further screened on formalin fixed paraffin embedded tissue sections. The initial screen was carried out on normal non-neoplastic human pancreas and blocks of the pancreatic tumour from which the GER cell line had originally been derived. Seven out of 14 stained pancreatic ducts and ductules in non-neoplastic formalin-fixed pancreatic tissue, and five of these also stained malignant epithelium in GER pancreatic tumour. Most of the antibodies had a similar pattern of staining and the supernatant which showed the most intense staining of malignant pancreatic epithelium (DD9E7) was produced as ascitic fluid in beige nude mice. These mice have defective macrophages (A. Sebesteny, personal communication) and therefore do not need to be pristane treated. The antibody was shown to be $IgG_{2b}$. Protein A purified immunoglobulin as well as culture supernatant was tested against other pancreatic adenocarcinomas (14), colorectal carcinomas (5), infiltrating ductular carcinomas of the breast (7) and a variety of non-neoplastic and malignant tissues. Twelve of the fourteen pancreatic adenocarcinomas showed strong staining of their

**Table II** Binding of monoclonals to a panel of gluteraldehyde-fixed tumour cells and normal human lymphocytes. Elisa assay using sheep anti-mouse urease conjugated antibody

O.D. at 588 nM

| *Cell line* / *Monoclonal* | *GER* | *WAD* | *HT29* | *AC* | *EC* | *RT4* | *GYL* | *MDA* | *HL* |
|---|---|---|---|---|---|---|---|---|---|
| 64. A-C6 | 0.12[a] | | 0.02 | | | | 0.04 | | 0 |
| 65. A-G4 | 0.18 | | 0.05 | | 0.12 | 0.09 | 0.09 | 0.08 | 0 |
| 65 A-G4-F5 | 0.06 | 0.13 | 0.14 | 0.11 | 0.08 | | 0.09 | 0.05 | 0.01 |
| 65. C-F11 | 0.07 | | 0.02 | 0 | | 0.01 | 0.01 | 0 | 0 |
| C-F11-F9-B7 | 0.03 | 0.10 | 0.11 | 0.08 | 0.06 | | 0.01 | | 0 |
| 65. D-D9 | 0.09 | | 0.06 | | 0.10 | 0.07 | 0.03 | 0.03 | 0 |
| D-D9-E7 | 0.10 | | 0.03 | 0.06 | | 0.07 | 0.01 | 0.05 | 0.01 |
| 65. F-E6/1 | 0.11 | | 0.06 | | 0.09 | 0.07 | 0.07 | 0.05 | 0.01 |
| F-E6/1-B8 | 0.08 | | 0.04 | 0.07 | | 0.07 | 0 | 0.03 | 0.02 |
| F-E6/1-C6 | 0.20 | | 0.05 | 0.07 | | 0.06 | 0.08 | 0.06 | 0.02 |
| F-E6/1-F4 | 0.17 | 0.12 | 0.09 | 0.11 | 0.08 | | 0.04 | | 0.05 |
| 65. F-E6/2 | 0.09 | | 0.01 | | 0.09 | 0.07 | 0.01 | 0.03 | 0 |
| 65. F-E10[b] | 0.06 | | 0.05 | 0.10 | | 0.06 | 0.09 | 0.10 | 0.04 |
| 67. C-B7 | 0.08 | 0.08 | 0.16 | 0.09 | 0.20 | | 0.05 | 0.02 | 0 |
| 67. D-G9 | 0.09 | 0.15 | 0.16 | 0.14 | 0.11 | | 0.11 | 0.07 | 0.04 |
| 67. F-D4 | 0.13 | 0.17 | 0.19 | 0.17 | 0.13 | | 0.13 | 0.06 | 0.07 |
| 67. F-D7[b] | 0.08 | 0.09 | 0.11 | 0.01 | 0.07 | | 0.04 | 0.01 | 0 |

[a]Positive wells had an OD < 0.07.
[b]Monoclonals did not bind to formalin fixed cells.

luminal membranes and cytoplasm (Figure 1), while included pancreatic islets were negative and acini and non-neoplastic ducts were often only weakly stained (Figure 2). In colorectal carcinomas, staining was mainly seen in the necrotic debris within acini while many luminal membranes were negative or only weakly stained (Figure 3). This was unlike the luminal pattern of staining found with a monoclonal against CEA (Figure 4). In 3/7 ductal carcinomas of the breast there was a grandular distribution of reaction product in the cytoplasm of a small population of tumour cells, quite unlike the luminal pattern of staining found with anti-EMA. DD9E7 also stained the supra-nuclear cytoplasm in some ducts and acini of normal human submandibular salivary gland and showed a luminal pattern of staining in normal colon and eccrine sweat glands. Except for included polymorphs and macrophages, which were stained in all sections, pituitary and placenta were negative, as were normal thyroid and a renal cell carcinoma. The immuno-cytochemical screening was also extended to cell lines which had been grown on glass slides prior to formalin fixation. The pancreatic cell line (GER), which was used as the immunogen, showed a heterogeneity of distribution with up to 70% of the cells showing a distinctive vesicular pattern of staining (Figure 5). In contrast, a fibrosarcoma HT1080 was completely negative (Figure 6).

### Discussion

A murine monoclonal antibody (DD9E7), with some discrimination for pancreatic adeno-carcinomas, has been generated by the fusion of NSO/1 cells with spleens from nude mouse hairy litter mates immunised with pancreatic tumour xenograft and cultured cells (GER). This method was considerably more successful than any of the other, more standard, immunisation routes in generating the highest proportion of supernatants (13%) which showed some selectivity for the target cell (GER). This may in part be due to the homology between the nude tumour bearing host and the immunised hairy litter mate, such that the immunological response is only against the human tumour cells rather than against the infiltrating mouse stroma. It is also possible that tumour cells growing *in vivo* as xenografts express antigens that are present on the original tumour but are lost on tissue culture, or that the transference of the mouse cell population of the tumour xenograft to a compatible immunocompetent host may contribute to the antibody response of the hairy litter mates.

Using the other methods only 4% of the hybrids produced antibody with selectivity for GER cells. Spleens from nude mice bearing tumour xenografts produced hybrids, despite their incomplete T cell system, but very few antibodies showed any selectivity. These results are similar to those obtained using tumour bearing immune-suppressed mice (Herlyn *et al.*, 1983). Spleens from hairy litter mates immunised with serum from GER tumour bearing mice were successful at producing antibody secreting hybrids, but again, the antibodies were relatively unselective.

The use of a modified ELISA assay made it possible to identify rapidly hybrid wells of potential usefulness for cloning and subsequent immuno-histochemical localisation studies. Glutaraldehyde fixation of the target cells was preferred to formaldehyde since many antigens were unstable on formalin fixation. The reactions found on glutaraldehyde fixation were also very similar to those present on viable cell monolayers and may more realistically reflect the antigen distribution *in vivo*. However, the ELISA assay was limited to the initial screening, since it could not allow for either the heterogeneity of the cell population or distribution of antigen on the cell surface. Immuno-histochemical staining of GER cells showed that ~70% of the cells exhibited antigen when the cells were approaching confluency in normal culture conditions, and the antigen had a vesicular pattern of distribution.

Fifty per cent of the antibodies which bound to GER cells were also reactive against antigens retained on formalin-fixed paraffin embedded tissue sections. This paralleled the loss of antigen activity found on formalin fixation of target cells in the ELISA assay. The most intensely staining monoclonal antibody, DD9E7, identified an antigen which was present in large amounts in 12/14 of pancreatic adenocarcinomas, absent from islets and variably expressed on normal acini ducts and ductules. The antigen was also found in colon and breast carcinomas and a number of normal tissues but its immunohistochemical pattern was different to that found with antibodies against CEA or EMA (Heyderman *et al.*, 1979; Grahame *et al.*, 1985). Consistent staining of polymorphs and macro-phages in all sections suggested the antigen may be an NCA-like material (Mach & Putztnaseri, 1972; Von Kleist *et al.*, 1972). The tissue distribution of the antigen together with preliminary studies, which show that DD9E7 binds to a component of ~55,00 mol. w in GER cell lysates (Winterbourne & Grant; unpublished observations), suggest that the antigen recognised by DD9E7 is unlike the sialo-ganglioside and mucin-like antigens recognised by CA19-9 (Magnani *et al.*, 1982; 1983) and Du-Pan-2 (Borowitz *et al.*, 1984).

**Figure 1** Moderately differentiated pancreatic adenocarcinoma stained with DD9E7 using an indirect immunoperoxidase technique. The cytoplasmic reactive cells are positive. ( × 125).

**Figure 2** In another field of the section shown in **Figure 1**, a residual pancreatic islet is negative, while residual non-neoplastic pancreatic ducts are weakly stained and a small focus of tumour (*bottom left*) is positive ( × 125).

**Figure 3** Moderately differentiated carcinoma of the colon stained with DD9E7. The staining is mainly in the necrotic debris, reactive polymorphs and macrophages in the stroma ( × 125).

**Figure 4** Another area of the same tumour as in **Figure 3** stained with a monoclonal antibody to CEA. The staining is mainly on the luminal membrane of malignant acini, and in the necrotic debris ( × 125).

**Figure 5** GER cell line grown on glass slide and stained with DD9E7. The distinctive vesicular pattern of staining in the cytoplasm is shown (×500).

**Figure 6** Fibrosarcoma cell line HT1080 also grown on slide and stained with DD9. No staining is seen (×500).

This study has shown that tumour cells growing *in vivo* as xenografts in nude mice are an ideal immunogen, when injected into syngenic relatives (nu/+), for the production of monoclonal antibodies to pancreatic tumour cells GER. One of these monoclonal antibodies, DD9E7, binds to an antigen present in pancreatic carcinomas. Its detection by DD9E7 may prove useful for both immunohistochemical and tumour localisation studies, and we are currently isolating the antigen for further biochemical analysis.

This work was supported by grants from the Cancer Research Campaign. We are most grateful to the ICRF and Dr A. Sebesteny for the supply of nude mice.

### References

ATKINSON, B.F., ERNST, C.S., HERLYN, M., STEPLEWSKI, Z., SEARS, H.F. & KOPROWSKI, A. (1982). Gastro-intestinal cancer associated antigens in immuno-peroxidase assay. *Cancer Res.*, **42**, 4820.

BOROWITZ, M.J., TUCK, F.L., SINDELAR, W.F., FERNSTEIN, P.D. & METZGAR, R.S. (1984). Monoclonal antibodies against human pancreatic adenocarcinoma: Distribution of Du-Pan-2 antigen on glandular epithelia and adenocarcinomas. *J. Natl Can. Inst.*, **72**, 999.

COBBOLD, S.P. & WALDMANN, H. (1981). A rapid solid-phase enzyme-linked binding assay for screening monoclonal antibodies to cell surface antigens. *J. Immunol. Meth.*, **44**, 125.

DAVIES, G., GRANT, A.G., DUKES, D. & HERMON-TAYLOR J. (1983). Antibody response of nude (RNU/RNU) and hairy (RNU/+) rats to circulating cell surface components from human pancreatic cancer xenografts. *Br. J. Cancer*, **48**, 239.

FOGH, J. & TREMPE, G. (1975). New tumour cell lines. In *Human Tumour Cells in vitro*, Fogh (ed) p. 115. Plenum Press, New York.

GALFRE, G. & MILSTEIN, C. (1981). Immunochemical Techniques Part B. In: *Methods in Enzymology*, **73**, 3.

GALFRE, G., HOWE, S.C., MILSTEIN, C., BUTCHER, G.W. & HOWARD, J.C. (1977). Antibodies to major histo-compatibility antigens produced by hybrid cell lines. *Nature*, **266**, 550–553.

GRAHAME, R.M., McKEE, P.H., CHAPMAN, D.V., RICHARDSON, T.V., STOKOE, M.R. & HEYDERMAN, E. (1985). Intercellular canaliculi in eccrine sweat glands. *Br. J. Dermatol.*, **221**, 397.

GRANT, A.G., DUKE, D. & HERMON-TAYLOR, J. (1979). Establishment and characterisation of primary human pancreatic carcinoma on continuous cell culture and in nude mice. *Br. J. Cancer*, **39**, 143.

GRANT, A.C. & DUKE, D. (1981). Production of antibodies against antigens released from human pancreatic tumour xenografts. *Br. J. Cancer*, **44**, 388.

HERLYN, D., ATKINSON, B. & KOPROWSKI, H. (1983). Monoclonal antibodies derived from immunosupressed mice grafted with human melanoma. *J. Immunol. Meths.*, **57**, 155.

HEYDERMAN, E. (1979). Immunoperoxidase technique in histopathology: Applications, methods and controls. *J. Clin. Path.*, **32**, 971.

HEYDERMAN, E., STRUDLEY, S.I., POWELL, G., RICHARDSON, T.C., CORDELL, J.L. & MASON, D.Y. (1985). A new monoclonal antibody to Epithelial Membrane Antigen(EMA)-E29. A comparison of its immunocytochemical reactivity with polyclonal anti-EMA antibodies and with another monoclonal antibody HMFG-2. *Br. J. Cancer*, **52**, 355.

HEYDERMAN, E. (1985). The immunoperoxidase technique in histopathology with special reference to malignant tumours of epithelial and germ-cell origin. MD Thesis, London University.

KOHLER, G. & MILSTEIN, C. (1975). Continuous cultures of fused cells secreting antibody of pre-defined specificity. *Nature*, **256**, 495.

MACH, J-P. & PUTZTNASERI, G. (1972). Carcino-embryonic antigen (CEA): Demonstration of a partial identity between CEA and a normal glycoprotein. *Immunochem.*, **9**, 1031.

MAGNANI, J.L., NILSSON, B., BROCKHAUS, M., ZOPF, D., STEPOWSKI, Z., KOPROWSKI, H. & GUISBURG, V. (1982). Monoclonal antibody defined antigen associated with gastrointestinal cancer is a ganglioside containing sialyated lacto-N-fucopentaose. *J. Biol. Chem.*, **257**, 14365.

MAGNANI, J.L., STEPLEWSKI, Z., KOPROWSKI, H. & GINSBERG, V. (1983). Identification of gastrointestinal and pancreatic cancer-associated antigen detected by monoclonal antibody 19-9 is the sera of patients as a mucin. *Cancer Res.*, **43**, 5489.

MATTHEWS, P.N., GRANT, A.G. & HERMON-TAYLOR, J. (1982). The growth of human bladder and kidney cancers as xenografts in nude mice and rats. *Urol. Res.*, **10**, 293.

MATTHEWS, P.N., HERMON-TAYLOR, J. & GRANT, A.G. (1984). An investigation of cellular components released from human renal cancer and foetal kidney xenografts in nude mice (nu/nu) by cross-immunisation of hairy litter-mate relatives. *Br. J. Cancer*, **49**, 193.

METZGAR, R.S., GAILLARD, M.T., LEVINE, S.J., TUCK, F.L., BOSSEN, E.H. & BOROWITZ, M.J. (1982). Antigens of human pancreatic adenocarcinoma cells defined by murine monoclonal antibodies. *Cancer Res.*, **42**, 601.

PARSA, I., SUTTON, A.L., CHEN, C.K. & DELBRIDGE, C. (1982). Monoclonal antibody for identification of human duct cell carcinoma of pancreas. *Cancer Lett.*, **17**, 217.

RASHEED, S., NELSON-REES, W.A., TOTH, E.M., ARBSTEIN, P. & GARDNER, M.B. (1974). Characterisation of a newly derived human sarcoma cell line (HT-1080). *Cancer*, **33**, 1027.

RIGBY, C.C. & FRANKS, L.M. (1970). A human tissue culture cell line from a transitional cell tumour of the urinary bladder: Growth, chromosome pattern and ultrastructure. *Br. J. Cancer*, **24**, 746.

SUTER, L., BRUGGEN, J. & SORG, C. (1980). Use of an enzyme-linked immunosorbent assay (ELISA) for screening of hybridoma antibodies against cell surface antigens. *J. Immunol. Meths.*, **39**, 407.

VON KLEIST, S., CHAVANEL, G. & BURTIN, P. (1972). Identification of an antigen from normal human tissue that cross-reacts with carcinoembryonic antigen. *Proc. Nat. Acad. Sci.*, **69**, 2492.

WALTON, J., WINTERBOURNE, D., FIENNES, A., HARRIS, P., HERMON-TAYLOR J. & GRANT, A.G. (1985). Human tumour cell lines established *in vitro* from tumour xenografts in nude animals. Comparative fingerprinting of their Con-A acceptor glycoproteins. *Br. J. Cancer*, **51**, 676.

YOUNG, R.K., CAILLEAU, R.M., MACKAY, B. & REEVES, W.J. (1974). Establishment of epithelial cell line MDA-MB-157 from metastatic pleural effusion of human breast carcinoma. *In vitro*, **9**, 239.